# The MoXFo Initiative: Using consensus methodology to move forward towards internationally shared vocabulary in multiple sclerosis exercise research

**DOI:** 10.1177/13524585231204460

**Published:** 2023-10-26

**Authors:** Maedeh Mansoubi, Yvonne Charlotte Learmonth, Nancy Mayo, Johnny Collet, Helen Dawes

**Affiliations:** Medical School, University of Exeter, Exeter, UK; NIHR Exeter Biomedical Research Centre, Medical School, Faculty of Health and Life Sciences, University of Exeter, Exeter, UK; INTERSECT, Medical School, University of Exeter, Exeter, UK; Discipline of Exercise Science, Murdoch University, Perth, WA, Australia; Centre for Molecular Medicine and Innovative Therapeutics, Healthy Futures Institute, Murdoch University, Perth, WA, Australia; Centre for Healthy Ageing, Healthy Futures Institute, Murdoch University, Perth, WA, Australia; Perron Institute for Neurological and Translational Science, Nedlands, WA, Australia; School of Physical & Occupational Therapy, McGill University, Montreal, QC, Canada; Centre for Movement, Occupational and Rehabilitation Science (MOReS), Oxford Brookes University, Oxford, UK; Medical School, University of Exeter, Exeter, UK; NIHR Exeter Biomedical Research Centre, Medical School, Faculty of Health and Life Sciences, University of Exeter, Exeter, UK; INTERSECT, Medical School, University of Exeter, Exeter, UK; Centre for Movement, Occupational and Rehabilitation Science (MOReS), Oxford Brookes University, Oxford, UK; Oxford Health Biomedical Research Centre, University of Oxford, Oxford, UK

**Keywords:** Vocabulary, lexicography, terminology, multiple sclerosis, rehabilitation, MoXFo

## Abstract

**Background:**

Multiple sclerosis (MS) exercise terminology lacks consistency across disciplines, hindering research synthesis.

**Objective:**

The ‘Moving exercise research in MS forward initiative’ (MoXFo) aims to establish agreed definitions for key MS exercise terms.

**Methods:**

The Lexicon development methodology was employed. A three-step process identified key exercise terminology for people with multiple sclerosis (pwMS): (1) consensus and systematic review, (2) Delphi round 1 and consideration of existing definitions and (3) Delphi round 2 for consensus among MoXFo steering group and exercise experts. Final definitions and style harmonisation were agreed upon.

**Results:**

The two-stage Delphi process resulted in the selection and scoring of 30 terminology definitions. The agreement was 100% for resistance exercise, balance and physical activity. Most terms had agreement >75%, but ‘posture’ (60%) and ‘exercise’ (65%) had a lower agreement.

**Conclusion:**

This study identified key terms and obtained agreement on definitions for 30 terms. The variability in agreement for some terms supports the need for clearly referencing or defining terminology within publications to enable clear communication across disciplines and to support precise synthesis and accurate interpretation of research.

## Introduction

The volume and quality of research on exercise interventions among people with multiple sclerosis (pwMS) have grown substantially in the last two decades.^1^ There is now strong evidence for the benefits of exercise in multiple sclerosis (MS).^2^ For people living with MS, exercise has the potential to improve physical fitness,^3,4^ walking mobility,^5^-^7^ strength,^7,8^ balance,^9^ cognition,^10^ fatigue,^4,11^-^13^ mood^14^ and quality of life.^15,16^ Evidence from interventions in pwMS further indicates that exercise improves outcomes measured by magnetic resonance imaging and modulates peripheral biomarkers associated with neural health.^17^ Exercise may benefit overall brain preservation,^18^-^20^ reduce relapse rate^21^ and might slow disability progression.^22^-^24^ However, exercise research studies in pwMS typically involve small sample sizes with a high diversity of outcome measures used, which limits research efficacy and the ability to pool data. A further issue, compounded by the interdisciplinary nature of exercise research in pwMS, is a lack of consensus on the vocabulary and definitions of these terms used in reporting the research. Terminology is used with varying meanings across disciplines. For example, the word intensity may be used to reflect the number of sessions or the metabolic equivalent of activities. Together, this limits the potency of reporting and comparison of intervention methodology, affecting the accuracy of interpretation and limiting the efficacy of subsequent systematic reviews and network meta-analyses.^25^

Recently, the ‘Moving exercise research in MS forward’ (MoXFo) initiative was established to address barriers to rapid progress in the field.^26^ The initial work of the MoXFo initiative identified five areas that needed attention including consensus work on definitions and terminology within the MS exercise field. Moreover, creating a consensus on vocabulary for use in future research studies is an initial step to supporting better methodological consistency and more precise interpretation across research studies and disciplines. Achieving consistency in the language used within and across scientific disciplines is challenging but a critical step in achieving research excellence and good practice. Importantly, consistency in health terminology has been shown to influence clinical studies positively and to create clarity in healthcare provider education,^27^ in translation to industry, and for pwMS.^28^

The scientific convention often calls for developing a standard lexicon across disciplines and geographical borders that underpins clear communication.^29^ This study followed a lexicon-development methodology,^29^ which has been successfully used to elevate clear communication both within and across professionals involved in interdisciplinary research in quality of life and health outcomes measurement literature in MS research.^25^ This project aimed to generate a vocabulary for MS exercise terminology for audiences with a specific set of needs (vertical audience), including people from the novice to the expert and from different disciplines, who all have a common interest in exercise prescription. We set out to develop a vocabulary to enable clear communication among MS researchers, MS healthcare and fitness professionals and pwMS and their families.^30^-^32^

## Method

The following approach was adopted. Initially, a group of researchers from the MoXFo initiative formed a steering group (the MoXFo steering group). The MoXFo initiative was initiated by U. Dalgas and C. Heesen, who gathered experts from five crucial areas of the field in a steering group. An author group for the actual terminology work was selected as having expertise in exercise and movement science, public health, rehabilitation, measurement methodology, Lexicography, neuroscience and physiology and representing North America, Australasia, West Asia and Europe.

All stages of the process were underpinned by Lexicography, a field methodology to create a vocabulary list of selected terms from the general language or a particular field of knowledge with brief definitions suitable for a vertical audience, which is one that includes those across disciplines, and from the novice to the expert.^29^ To achieve this, the following methodologies were used: a literature review, a consultation with professional bodies, an informal consensus development panel, a two-stage Delphi process and a final definitions and style harmonisation consensus development panel. The project plan and timeline outlining the aims, methods and analysis were developed by M.M. and H.D. with oversight of all authors (M.M., J.C., Y.C.L., H.D. and N.M.). The lexicon methodology^25^ employed in this study involved a series of steps to determine and provide clear definitions for key terminology related to exercise in MS.

**Figure 1.**
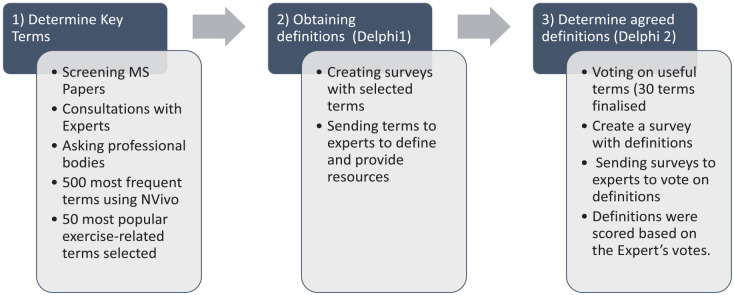
Flow diagram showing the different stages of the project.

In this study, a group of 30 professionals in the field, along with a systematic review of exercise in pwMS, were used to identify appropriate terminology related to exercise in PWMS. The selected terminology then underwent a process of finding definitions, which involved 24 experts and consideration of available definitions from professional bodies (Delphi 1). Finally, a consensus methodology approach was used, which involved the MoXFo steering group and 24 exercise experts, to agree on the definitions of each term. This was achieved through a Delphi 2 round, where the experts provided their feedback and opinions on the proposed definitions, leading to a consensus on the terminology and its definition for use in future studies related to exercise in PWMS. The authors and the MoXFo steering group carried out a final harmonisation of term definitions. A flow diagram showing the different stages of the process is depicted in Figure 1.

The detailed process of the three stages of the project is comprehensively explained below.

### Determine key terms

(1) To determine common usage in clinical trials, we used all the full text of 81 articles from a systematic literature review produced by the MoXFo exercise trials reporting and outcomes group.^33^ NVivo AI software (QRS) identified the 500 most frequently used terms from the full text of all 81 manuscripts. NVivo is a computer-assisted qualitative data analysis software (CAQDAS) that aids researchers in organising, analysing and visualising unstructured or semi-structured data. One of the key features of NVivo is its ability to identify and extract the most common terms in a document or a set of documents. This process, called text mining, enables researchers to identify patterns and trends in large data sets that may be difficult to detect through manual analysis.

After the data mining process of all the articles using NVivo, the list included terms not related to exercise and physical activity. M.M. and H.D. searched the list by hand and identified the 50 most frequently used terms related to exercise and physical activity (stage a).^33^

(2) Simultaneously, an expert panel that included the authors, the MoXFo steering group, and 30 International experts, representing disciplines of physical activity, physiotherapy and exercise science/physiology and different geographical regions were invited to identify key terms. Each expert was asked to identify 10 key terms that required definition clarity (Supplemental Appendix 2). Criteria for the inclusion of experts were that members must be researchers or professionals working in physical activity or clinical exercise science or with expertise in exercise science and MS. Identification of appropriate panellists were performed through the manual review of relevant peer-reviewed literature, personal networks and special interest groups for physiotherapy, physical activity and exercise science/physiology. Twenty-four experts agreed to participate. Participating experts were asked to identify 10 key terms from the 50 most frequently used terms. Each in relation to exercise and MS, requiring definition clarity.
(3) Duplicates were removed, and terms from the review (42 terms) and experts (8 additional terms) were merged. A total number of 50 terms were left.

### Obtaining definitions from the selected key terminology (Delphi 1)

(1) Two researchers (H.D. and Y.C.L.) approached professional bodies (physical activity/public health, exercise science/physiology, physiotherapy) from English-speaking nations to collect already available definitions and vocabulary (Supplemental Appendix 2). Utilising Delphi methodology to determine consensus, initially, a questionnaire (Delphi 1) was simultaneously sent out to the 24 experts to determine definitions for all 50 terms. The literature makes no formal recommendation regarding the number of survey voting (Delphi) members; above 8 is considered acceptable, but 20 is common.^34^ The questionnaire was piloted on five individuals to test functionality and clarity, which was then confirmed through the study panel. Each expert was requested to define five terms independently. Each term was sent to at least two experts, so there was cross-over. Terms were matched to experts considering their expertise (used references and resources are listed in Supplemental Appendix 1). A resulting long list of vocabulary and definitions from experts and professional bodies was progressed to the next stage.^34,35^ Terms were considered out of scope and not included in the survey if they were biomarkers, adherence or other non-exercise focus terms and reduced down to 30 terms.^36^

### Determine agreed definitions for selected terms (Delphi 2)

Two definitions were allocated for each term in order to reduce burden or ranking. Where there were more than two definitions, for example, when both experts and an expert body definition were available, the authors (M.M., J.C., Y.C.L., H.D. and N.M.) met as an informal consensus development panel to select two definitions. For the second round of Delphi (Delphi 2), each term with two associated definitions were then sent to at least two people from the 24 experts for voting. Where there was a strong authority definition from a professional body and agreed upon by the authors, this was selected. In this instance, the one definition was sent out with a yes/no question.

All highest-ranked definitions based on the expert’s votes were then editorially harmonised for style by the authors (M.M., J.C., Y.C.L., H.D. and N.M.) in a consensus meeting and later by final editorial by the senior author in exchange with the MoXFo steering group. Final editorial harmonisation was carried out for consistency in the structuring of terminology definitions.

## Data analysis

Frequency and descriptive analysis were completed on the responses in the Delphi 2. The agreement was carried out by the non-parametric assessment; Kendall’s *W* coefficient of concordance was used to quantify the extent of agreement between raters.^37^ Data were analysed in SPSS version 28. An a priori arbitrary cut point was set so that all terms with >50% agreement were included. All terms are listed with agreed and non-agreed terminology in Supplementary Material.

## Reducing bias

To address issues of both implicit and explicit bias, we recruited experts across disciplines and geography, and across both the MS scientific community and with wider clinical reach. We found the most cited authors and approached them, and then used a snowball technique to other experts. We also followed personal contacts through the MoXFo steering group. At all stages, teams worked together with different backgrounds and disciplines. Experts were paired to maximise diversity in geography and background. Critical stages were carried out by a consensus development panel (M.M., J.C., Y.C.L., H.D. and N.M.), and the final consensus was taken to the MoXFo steering group for agreement.

## Results

Data on key terms were gathered between 15 August and 15 September 2021.

Key terminology selection: the review included 81 full papers from which the initial top 500 most frequently used terms were ranked, of which the top 50 exercise terms were taken forward to the next stage. Experts independently highlighted 15 terms for inclusion; all these terms were in the top 50 set of terms from the review.
Definition determination: 50 terms were sent out for definitions. The terms, frequency and relevance data are available from the authors on request. If the terms were biomarkers, adherence or other non-exercise-related terms, they were excluded from the survey as they were considered out of scope. The number of terms was then reduced to 30. The authors were able to use 30 terms in Delphi 2, considering the described criteria, with associated definitions that were then sent out for consensus (Tables 1 and 2). Table 1 summarises the terms and definitions.
Consensus methods: 28 term definitions achieved over 75% agreement. Definitions with scoring are presented in (Table 1). The final agreed definitions from the consensus process and following subsequent harmonisation process are listed in Tables 1 and 2 and Figure 1.

**Table 1.** Terms definitions scores, based on the experts’ votes.

Word	Top-voted definition number of votes	Score (%)
1. Aerobic exercise	16/20	80
2. Anaerobic exercise	16/20	80
3. Balance	20/20	100
4. Core stability	19/20	95
5. Endurance (exercise)	19/20	95
6. Exercise	13/20	65
7. Exercise capacity	15/20	75
8. Exercise intensity	16/20	80
9. Fatigue	17/20	85
10. Central fatigue/perception of fatigue	17/20	85
11. Peripheral fatigue/fatigability	17/20	85
12. Physiological fatigue (fatiguability)	16/20	80
13. Mental fatigue	17/20	85
14. Flexibility	16/20	80
15. Frequency	19/20	95
16. High-intensity interval training (HIIT)	19/20	95
17. Maximal oxygen consumption (VO_2_ max)	19/20	95
18. Mobility	15/20	75
19. Motor learning	17/20	85
20. Performance	15/20	75
21. Physical activity	20/20	100
22. Physical fitness	14/20	70
23. Posture	12/20	60
24. Power	18/20	90
25. Prescription (in physical activity)	17/20	85
26. Resistance exercise	20/20	100
27. Sedentary behaviour	18/20	90
28. Self-efficacy	17/20	85
29. Strength	17/20	85
30. VO_2_ peak	19/20	95

HIIT: high-intensity interval training.

**Table 2.** Final definitions (see the Supplemental Appendix 1 for references and resources that have been used for providing the definitions).

Word	
1. Aerobic exercise	Any activity that uses large muscle groups, can be maintained continuously and is rhythmic in nature. Muscle groups activated by this type of exercise rely predominantly on aerobic metabolism to extract energy. Examples of aerobic exercise include cycling, dancing, hiking, jogging/long distance running, swimming and walking.
2. Anaerobic exercise	Intense physical activity of short duration, predominantly fuelled by the energy sources within the contracting muscles and independent of the use of inhaled oxygen as an energy source.
3. Balance	An individual’s ability to maintain their line of gravity within their base of support; also, the ability to maintain equilibrium, a condition in which actions are successfully engaged to cancel perturbating forces.
4. Core stability	The ability of the segments of the body to remain aligned during physically demanding tasks involving big movements or torque generation; segmental stability is achieved as a result of the engagement, voluntary or involuntary, of the stabilising muscles, including deep/local muscles (e.g. transversus abdominis, lumbar multifidus) and/or the superficial/global muscles (e.g. rectus abdominis, erector spinae).
5. Endurance (exercise)	The individual’s ability to perform the specific activity of physical tasks for periods of time. Endurance exercise is also referred to as aerobic exercise. See definition for aerobic exercise.
6. Exercise	Physical activity that is planned, structured, repetitive and for the purpose of improvement or maintenance of one or more components of physical fitness.
7. Exercise capacity	Exercise capacity is the maximum amount of physical exertion that a person can sustain. Exercise capacity is usually quantified as the ability of an individual to perform aerobic work as defined by the maximal oxygen uptake (V̇o_2 max_) (see definition for V̇o_2 max_). It is the product of cardiac output and arteriovenous oxygen (a − V̇o_2_) difference at physical exhaustion. An accurate assessment of exercise capacity requires that maximal exertion is sufficiently prolonged to have a stable (or steady state) effect on the circulation. This is not always possibly in clinical populations. Exercise capacity is the product of the capacity of the cardiorespiratory system to supply oxygen and the capacity of the skeletal muscles to utilise oxygen.
8. Exercise intensity	The amount of energy required for the performance of the physical activity per unit of time. This can be measured directly using respiratory gas analysis to quantify oxygen uptake during exercise or can be estimated using standard regression models to estimate energy expenditure per given work rate of exercise. Exercise intensity can also be expressed in terms of resting oxygen requirement (metabolic equivalents (METs)), where one MET equals the amount of oxygen consumed by a resting, awake individual and is equivalent to 3.5 mL O_2_/kg of body weight/minute. Light exercise denotes those activities requiring 1.5 to 3 METs, moderate activity denotes activities requiring >3 to 6 METs (moderate exercise intensity: 50% to about 70% of individual maximum heart rate), and vigorous activity denotes activities requiring more than 6 METs (vigorous exercise intensity: 70% to about 85% of individual maximum heart rate). Other methods that can be used to estimate intensity include the use of heart rate reserve.
9. Fatigue	Difficulty in the initiation or sustaining voluntary activities which can be distinguished from the lay notion of tiredness. Perception of fatigue and performance fatigability are two components of fatigue that have different causes, manifestations and life impacts. There is no single agreed-upon taxonomy for classifying fatigue. The following terms are used, albeit inconsistently, in the literature to refer to fatigue
10. Central fatigue/perception of fatigue	A feeling of constant exhaustion typically not improved by rest. Central fatigue may be caused by wide-ranging pathology mechanisms and is regulated by central and autonomic nervous systems. Physiological and psychological stimuli produce.
11. Peripheral fatigue/fatigability	Muscle fatigability due to disorders of muscle and neuromuscular junction; often restored at least partially by rest.
12. Physiological fatigue (fatiguability)	Occurs if exercise continues to the point where muscle glycogen is depleted. It is commonly measured by physiological or performance tests on muscles. Physiological fatigue is only one component affecting initiation of or sustaining voluntary activities (work output) which is also influenced by cognitive and sensory factors, perceived exertion, motivation and incentives and is also influenced by homeostatic (endocrine and autonomic) factors and the external environments (such as temperature).
13. Mental fatigue	The cognitive component of central fatigue is characterised by the inability to sustain concentration and endure mental tasks as distinct from apathy to initiate activity. It is typically assessed by PROs or by measuring deterioration in cognitive performance on tests of cognitive processing administered over time (typically several hours).
14. Flexibility	The extent a joint or series of joints can move through a needed, range of motion.
15.Frequency	A count of the number of occurrences, events or repetitions observed in a population or experimental session. In the context of physical activity, it is common to count the frequency of activity bouts over a discrete period of time such as 10 walking bouts per day.
16. High-intensity interval training (HIIT)	Involves repeated bouts of high-intensity effort followed by various recovery times. The intense work periods may range from 5 seconds to 8 minutes long and are performed at 80%–95% of a person’s estimated maximal heart rate, the maximum number of times the heart will beat in a minute without overexerting the body. The recovery periods may last equally as long as the work periods and are usually performed at 40%–50% of a person’s estimated maximal heart rate. The workout continues with alternating work and relief periods totalling 20–60 minutes.
17. Maximal oxygen consumption (VO_2_ max)	The greatest amount of oxygen a person can take in from inspired air per unit of time while performing dynamic exercise involving a large part of total muscle mass. ˙It is the amount of oxygen transported and utilised for cellular metabolism and is the product of the cardiac output and the arteriovenous O_2_ difference. It is considered the best measure of cardiovascular fitness and exercise capacity.
18. Mobility	The changing and maintaining of body position, carrying, moving and handling objects, walking and moving around using transportation. It has also been defined in rehabilitation ‘as the ability to move oneself (either independently or using assistive devices or transportation) within environments that expand from one’s home to the neighbourhood and to regions beyond’.
19. Motor learning	The process of the acquisition and/or modification of skilled action. In essence, it is the process of learning how to do something well.
20. Performance	The execution of a task or activity in the person’s usual environment in contrast to capacity which is in a standard environment, such as a clinic or laboratory. In rehabilitation, it is also used to refer to assessment that requires a person to ‘perform’ a test, such as the six-minute walk test (6MWT); these types of tests are termed Performance Outcomes (PerfO). Within the ICF framework, a PerfO would be considered a test of capacity, whereas a test of performance would be whether the person walks outside in their community or around their house.
21. Physical activity	Physical activity includes any bodily movements produced by skeletal muscle contraction resulting in increased energy expenditure. Physical activity broadly encompasses exercise, sports and physical activities done as part of daily living, occupation, leisure and active transportation.
22. Physical fitness	‘The ability to carry out daily tasks with vigour and alertness, without undue fatigue and with ample energy to enjoy [leisure] pursuits and to meet unforeseen emergencies’. Physical fitness is operationalised as ‘[a set of] measurable health and skill-related attributes’ that include cardiorespiratory fitness, muscular strength and endurance, body composition and flexibility, balance, agility, reaction time and power.
23. Posture	The position in which the body is held owing to musculoskeletal structures and/or muscular activity.
24. Power	The amount of mechanical work performed over a discrete period. It is measured in Watts which equates to 1 Joule of work per second.
25. Prescription (in physical activity)	The explicit instructions for exercise that are specific as to the type, intensity, frequency and duration of activity to be performed.
26. Resistance exercise	A type of exercise that involves the muscles contracting against an external force (weights, resistance bands, one’s body weight, gravity). Resistance exercise is also known as strength or weight training.
27. Sedentary behaviour	‘Any waking behaviour characterised by an energy expenditure of ⩽1.5 METs while in a sitting or reclining posture.’
28. Self-efficacy	Self-efficacy refers to people’s beliefs in their capability to exercise control over their own functioning and over events that affect their lives. Self-efficacy beliefs determine how people feel, think, motivate themselves and behave.
29. Strength	The capacity of a muscle to generate force. Muscle activity can be classified by isometric (muscle length does not change during contraction) and isotonic muscle force. Isotonic force can be further described as concentric muscle activity when force is generated when the muscle is shortening, and eccentric muscle activity is force generation when the muscle is lengthening.
30. VO_2_ peak	The value maximally attained during a VO_2_ max testing should be reported as the subject’s VO_2_ peak when there is no demonstrable evidence that the criterion for VO_2_ max has been met in an exercise test (See VO_2_ max).

HIIT: high-intensity interval training; 6MWT: six-minute walk test; ICF: International Classification of Functioning, Disability and Health.

## Discussion

Here, we set out to consider the terminology and definitions for core exercise terms and move towards internationally shared vocabulary in MS exercise research for exercise scientists, clinicians and pwMS and those involved in their care. In this study, we established majority agreement in vocabulary for 30 terms. There was complete (100%) agreement from the expert reviewers in the three terms’: resistance exercise, balance and physical activity. There was good agreement (>75%) in the majority of terms, with the lowest agreement for the terms ‘posture’ (60%) and ‘exercise’ (65%). However, there was still extensive discussion at the consensus development and the MoXFo steering group panels on the final agreed terminologies. These discussions included debates about the nuances of specific terms, such as their definitions and appropriate usage, as well as considerations of the cultural and linguistic differences that might affect their interpretation. In addition, there were also discussions around the inclusion or exclusion of certain terms, with some experts arguing for the inclusion of additional terms to provide a more comprehensive vocabulary for MS exercise research. Ultimately, after careful consideration of all viewpoints and extensive deliberation, we propose that this paper provides initial information about definitions to be used, but that further systematic methodology is needed to identify comprehensive definitions for the field.

MS is a complex neurological disorder affecting millions worldwide and involving multidisciplinary teams. Unfortunately, this has resulted in a lack of unified terminology and underlying definitions, leading to confusion, miscommunication and misunderstandings. Having clear definitions for terms makes it easier for healthcare professionals to communicate with each other and patients. It also ensures that pwMS receive accurate information about their condition. Without a unified terminology, healthcare professionals may use different terms to describe the same concept, leading to imprecise treatment and communication. Hence, we recommend that researchers clearly reference or state definitions of their terms so that data can be appropriately combined in meta-analyses. There were some notable important gaps, where we were unable to recommend terminology, such as anaerobic capacity and intensity, where further work is needed. It was also hard to agree when fundamental differences existed, such as the observed difference in understanding of the word ‘intensity’ across clinical and research teams. We propose that this important fundamental work still needs to be performed.

Our approach has limitations, whereby we set out to harmonise terminology across a range of disciplines, with completely different training backgrounds, which led to some disagreement in the level of detail required, or definitions that should be used. There is inevitable bias in the inclusion of experts, although we attempted to invite experts across a wide geography and expertise. We used a final harmonisation process, which inevitably led to some bias in decision-making, although a consensus panel, including the MoXFo steering group, was used for the final agreement.

While considering the limitations in our approach, we do propose that there is an urgent need for researchers to include literature in systematic reviews and meta-analyses with confidence that they are synthesising appropriate interventions and measures. This is hard to do when fundamental differences exist, such as the observed difference in understanding of the word ‘intensity’ across clinical and research teams. There will be a number of terms that are not currently in common usage and new terms that will appear as the area evolves, and we propose that researchers proactively define these terms in research papers. We request that editors encourage that terminology is either clearly defined or referenced in order to increase confidence in the interpretation of research findings and research efficacy in this area.

## Supplemental Material

sj-docx-1-msj-10.1177_13524585231204460 – Supplemental material for The MoXFo Initiative: Using consensus methodology to move forward towards internationally shared vocabulary in multiple sclerosis exercise research Click here for additional data file. Supplemental material, sj-docx-1-msj-10.1177_13524585231204460 for The MoXFo Initiative: Using consensus methodology to move forward towards internationally shared vocabulary in multiple sclerosis exercise research by Maedeh Mansoubi, Yvonne Charlotte Learmonth, Nancy Mayo, Johnny Collet and Helen Dawes in Multiple Sclerosis Journal

sj-docx-2-msj-10.1177_13524585231204460 – Supplemental material for The MoXFo Initiative: Using consensus methodology to move forward towards internationally shared vocabulary in multiple sclerosis exercise research Click here for additional data file. Supplemental material, sj-docx-2-msj-10.1177_13524585231204460 for The MoXFo Initiative: Using consensus methodology to move forward towards internationally shared vocabulary in multiple sclerosis exercise research by Maedeh Mansoubi, Yvonne Charlotte Learmonth, Nancy Mayo, Johnny Collet and Helen Dawes in Multiple Sclerosis Journal
