# Supplementary material for: The MoXFo Initiative: Using consensus methodology to move forward towards internationally shared vocabulary in multiple sclerosis exercise research
Source: Mult Scler. 2023 Oct 26;29(13):1551–60. doi: 10.1177/13524585231204460 (PMC10637107; doi:10.1177/13524585231204460)
Supplement: sj-docx-1-msj-10.1177_13524585231204460 – Supplemental material for The MoXFo Initiative: Using consensus methodology to move forward towards internationally shared vocabulary in multiple sclerosis exercise research [file sj-docx-1-msj-10.1177_13524585231204460.docx]

Appendix 1: Reference and resources that have been used for defining the terms are listed below:

Patel H, Alkhawam H, Madanieh R, Shah N, Kosmas CE, Vittorio TJ. (2017). Aerobic vs anaerobic exercise training effects on the cardiovascular system. World J Cardiol. 9(2):134-138. doi:10.4330/wjc.v9.i2.134

Healthline Anaerobic Exercise Available from: https://www.healthline.com/health/fitness-exercise/anaerobic-exercise#types (accessed 20.05.2022)

Patel H, Alkhawam H, Madanieh R, Shah N, Kosmas CE, Vittorio TJ. (2017) Aerobic vs anaerobic exercise training effects on the cardiovascular system. World J Cardiol. 9(2):134-138. doi:10.4330/wjc.v9.i2.134

American College of Sports Medicine. ACSM's Resource Manual for Guidelines for Exercise Testing and Prescription. 6th edition ed. Baltimore, MD: Lippincott Williams & Wilkins; 2010.

Physiopedia. https://www.physio-pedia.com/Anaerobic_Exercise#:~:text=Anaerobic%20exercise%20is%20any%20activity,short%20length%20with%20high%20intensity.(accessed 20.5.2022)

Physiopedia. https://www.physio-pedia.com/Balance#cite_ref-susan_1-0. (accessed 20.05.2022)

Studnicka K, Ampat G. Lumbar Stabilization. [Updated 2021 Nov 29]. In: StatPearls [Internet]. Treasure Island (FL): StatPearls Publishing; 2022 Jan-. Available from: https://www.ncbi.nlm.nih.gov/books/NBK562179/

Dishman RK, Sallis JF, Orenstein DR. (1985) The determinants of physical activity and exercise. Public Health Rep. 100(2):158-171.

Caspersen CJ, Powell KE, Christenson GM.(1985) Physical activity, exercise, and physical fitness: definitions and distinctions for health-related research. Public Health Rep. 100(2):126-131.

Nancy Mayo, ISOQOL Dictionary of Quality of Life and Health Outcomes Measurement. 2015

Goldstein RE. Clinical Methods: The History, Physical and Laboratory Examinations. (2002). Third Edition ed. Boston: Buttersworth; 1990. 130. Myers J, Prakash M, Froelicher V, Do D, Partington S, Atwood JE. Exercise capacity and mortality among men referred for exercise testing. N Engl J Med 2002;346(11):793-801. 131.

Kaminsky DA, Knyazhitskiy A, Sadeghi A, Irvin CG. (2014). Assessing maximal exercise capacity: peak work or peak oxygen consumption? Respir Care 2014;59(1):90-96. 132.

Padulo, J., Laffaye, G., Chamari, K., & Concu, A. (2013). Concentric and eccentric: muscle contraction or exercise?. Sports health, 5(4), 306. https://doi.org/10.1177/1941738113491386

Boston P, Bruce A, Schreiber R. (2011). Existential suffering in the palliative care setting: an integrated literature review. J Pain Symptom Manage. 41(3):604-618.

Arena R, Myers J, Williams MA, et al.(2007) Assessment of functional capacity in clinical and research settings: a scientific statement from the American Heart Association Committee on Exercise, Rehabilitation, and Prevention of the Council on Clinical Cardiology and the Council on Cardiovascular Nursing. Circulation. 116(3):329-343. doi:10.1161/CIRCULATIONAHA.106.184461

Hills AP, Mokhtar N, Byrne NM. (2014). Assessment of physical activity and energy expenditure: an overview of objective measures. Front Nutr. 2014;1:5. Published 2014 Jun 16. doi:10.3389/fnut.2014.00005

Thompson WR, Gordon NF, Pescatello LS. ACSM’s Guidelines for Exercise Testing and Prescription. 8th ed. Philadelphia (Pa): Lippincott Williams & Wilkins; 2010. p. 453

Meeusen R, Watson P, Hasegawa H, Roelands B, Piacentini MF. (2006). Central fatigue: the serotonin hypothesis and beyond. Sports Med. 2006;36(10):881-909. doi: 10.2165/00007256-200636100-00006. PMID: 17004850.

Davis JM, Bailey SP. (1997). Possible mechanisms of central nervous system fatigue during exercise. Med Sci Sports Exerc. 1997 Jan;29(1):45-57. doi: 10.1097/00005768-199701000-00008. PMID: 9000155.

Davis JM, Bailey SP. (1997). Possible mechanisms of central nervous system fatigue during exercise. Med Sci Sports Exerc. 1997 Jan;29(1):45-57. doi: 10.1097/00005768-199701000-00008. PMID: 9000155.

Meeusen R, Watson P, Hasegawa H, Roelands B, Piacentini MF. (2006). Central fatigue: the serotonin hypothesis and beyond. Sports Med. 2006;36(10):881-909. doi: 10.2165/00007256-200636100-00006. PMID: 17004850.

Booth FW, Roberts CK, Laye MJ. (2012). Lack of exercise is a major cause of chronic diseases. Compr Physiol. 2012;2(2):1143-1211. doi:10.1002/cphy.c110025

American College of Sprot Medicine. https://www.acsm.org/docs/default-source/files-for-resource-library/high-intensity-interval-training.pdf (Accessed 25/03/2022)

Raghuveer G, Hartz J, Lubans DR, Takken T, Wiltz JL, Mietus-Snyder M, Perak AM, Baker-Smith C, Pietris N, Edwards NM. (2020) Cardiorespiratory Fitness in Youth: An Important Marker of Health: A Scientific Statement From the American Heart Association. Circulation. 2020;142(7):e101-e118. doi:10.1161/CIR.0000000000000866

International Classification of Functioning, Disability and Health (ICF). https://www.who.int/standards/classifications/international-classification-of-functioning-disability-and-health

Webber SC, Porter MM, Menec VH. (2010) Mobility in older adults: a comprehensive framework. Gerontologist 2010 Aug;50(4):443-50.)

Webber SC, Porter MM, Menec VH.(2010). Mobility in older adults: a comprehensive framework. Gerontologist 2010 Aug;50(4):443-50.

Sattelmayer M, Elsig S, Hilfiker R, Baer G. (2016). A systematic review and meta-analysis of selected motor learning principles in physiotherapy and medical education. BMC Med Educ. 2016; 16(15).

Bisson T. Motor Learning - Back to Basics Course. Physioplus. 2020

Garber CE, Blissmer B, Deschenes MR, et al. (2011) American College of Sports Medicine position stand. Quantity and quality of exercise for developing and maintaining cardiorespiratory, musculoskeletal, and neuromotor fitness in apparently healthy adults: guidance for prescribing exercise. Med Sci Sports Exerc.43(7):1334-1359. doi:10.1249/MSS.0b013e318213fefb

Physipedia. https://www.physio-pedia.com/Posture (Accessed 25/03/2022)

National Research Council (US) and Institute of Medicine (US) Panel on Musculoskeletal Disorders and the Workplace. Musculoskeletal Disorders and the Workplace: Low Back and Upper Extremities. Washington (DC): National Academies Press (US); 2001. 6, Biomechanics. Available from: https://www.ncbi.nlm.nih.gov/books/NBK222434/

Gordis L. Epidemiology. 3rd ed. Philadelphia, PA: Elsevier Saunders; 2004.

Resistance training – health benefits. Victoria state government. Department of health. https://www.betterhealth.vic.gov.au/health/healthyliving/resistance-training-health-benefits

Network SBR. Letter to the editor: standardised Use of the terms "sedentary" and "sedentary behaviours". Appl Physiol Nutr Metab. 2012;37:540–2.

314 Bandura A. Self-efficacy: The exercise of control. New York: W.H. Freeman; 1997.

WHO Glossary of terms WHO European Primary Health Care Impact, Performance and Capacity Tool (PHC-IMPACT) https://www.euro.who.int/__data/assets/pdf_file/0006/421944/Glossary-web-171219.pdf
