# Supplementary material for: The MoXFo Initiative: Using consensus methodology to move forward towards internationally shared vocabulary in multiple sclerosis exercise research
Source: Mult Scler. 2023 Oct 26;29(13):1551–60. doi: 10.1177/13524585231204460 (PMC10637107; doi:10.1177/13524585231204460)
Supplement: sj-docx-2-msj-10.1177_13524585231204460 – Supplemental material for The MoXFo Initiative: Using consensus methodology to move forward towards internationally shared vocabulary in multiple sclerosis exercise research [file sj-docx-2-msj-10.1177_13524585231204460.docx]

**International Expert:** This study would like to say a special thanks to a few of the study experts who helped us with their scientific views; "Dr Sina Cathérine Rosenkranz, Dr Gavin Williams, Dr Andrea Tacchino, Dr Moira Smith, Dr Paul Goods, Dr Daphne Kos, Dr Priyankara Manoj Rajakaruna Rajakaruna Mudiyanselage, Dr Veronica Varela Mato, Dr Ender Ersin Avcı, Dr Kedar K. V. Mate, Ludovico Pedullà, Dr James Sanders" and other experts which preferred to remain unknown. Thank you for your support and for making this important endeavour possible.

**Moxfo steering group:**

Helen Dawes, Ulrik Dalgas, Chris Heesen, Rob Motl, Lorna Paul, Manuel Friese, Sina Rosenkranz, Lars G. Hvid

**Professional Bodies:**

Exercise Sports Science Australia (ESSA),

American College of Sports Medicine (ACSM),

Sport and Exercise Science New Zealand (SESNZ),

European College of Sports Science (ECSS),

British Association of Sport and Exercise Sciences (BASES),

South African Sports Medicine Association (SASMA),

Canadian Academy of Sports and Exercise Medicine (CASEM)

Australian Physiotherapy Association (APA), American Physical Therapy Association (APTA), Physiotherapy Board of New Zealand (PNZ), Chartered Society of Physiotherapy (CSP), South African Society of Physiotherapy (SASP), Canadian Physiotherapy Association (CPA)

World Health Organization (WHO)

Association for Applied Sports Psychology (AASP)

Clinical Exercise Physiology Association (CEPA)

The Canadian Society for Exercise Physiology (CSEP)

International Society for Physical Activity and Health (ISPAH)

American Medical Association

American Physical Therapy Association (APTA)

European college of sport sciences (ECSS)
